# Transmembrane Protein 68 Functions as an MGAT and DGAT Enzyme for Triacylglycerol Biosynthesis

**DOI:** 10.3390/ijms24032012

**Published:** 2023-01-19

**Authors:** Yu Wang, Fansi Zeng, Zheng Zhao, Lin He, Xiaohong He, Huimin Pang, Feifei Huang, Pingan Chang

**Affiliations:** Chongqing Key Laboratory of Big Data for Bio-Intelligence, School of Bio-Information, Chongqing University of Posts and Telecommunications, Chongqing 400065, China

**Keywords:** triacylglycerol biosynthesis, monoacylglycerol acyltransferase, diacylglycerol acyltransferase, lipid droplet, transmembrane protein 68

## Abstract

Triacylglycerol (TG) biosynthesis is an important metabolic process for intracellular storage of surplus energy, intestinal dietary fat absorption, attenuation of lipotoxicity, lipid transportation, lactation and signal transduction in mammals. Transmembrane protein 68 (TMEM68) is an endoplasmic reticulum (ER)-anchored acyltransferase family member of unknown function. In the current study we show that overexpression of TMEM68 promotes TG accumulation and lipid droplet (LD) formation in a conserved active sites-dependent manner. Quantitative targeted lipidomic analysis showed that diacylglycerol (DG), free fatty acid (FFA) and TG levels were increased by TMEM68 expression. In addition, TMEM68 overexpression affected the levels of several glycerophospholipids, such as phosphatidylcholine, phosphatidylethanolamine and phosphatidylinositol, as well as sterol ester contents. TMEM68 exhibited monoacylglycerol acyltransferase (MGAT) and diacylglycerol acyltransferase (DGAT) activities dependent on the conserved active sites in an in vitro assay. The expression of lipogenesis genes, including DGATs, fatty acid synthesis-related genes and peroxisome proliferator-activated receptor γ was upregulated in TMEM68-overexpressing cells. These results together demonstrate for the first time that TMEM68 functions as an acyltransferase and affects lipogenic gene expression, glycerolipid metabolism and TG storage in mammalian cells.

## 1. Introduction

Triacylglycerol (TG) plays critical roles in various physiological processes, such as intracellular surplus energy storage, intestinal dietary fat absorption, lipotoxicity attenation, lactation, lipid transportation and lipid-mediated signal transduction in mammals [1,2]. Synthesis of TG is a fundamental metabolic pathway. During conditions of fatty acid (FA) surplus, TG synthesis is promoted and stored in organelles termed lipid droplets (LDs) [3,4]. There are two main biochemical pathways for TG biosynthesis in mammals, the monoacylglycerol (MG) pathway and the glycerol 3-phosphate (G-3-P) pathway [1,2].

In the MG pathway, the first enzymatic step is the acylation of MG with acyl-CoA to diglyceride (DG) catalyzed by MG acyltransferase (MGAT) [1,2]. DG is then further acylated to TG by DG acyltransferase (DGAT) [1,2]. This pathway serves a predominant function in enterocyte TG synthesis after feeding and contributes ~75% of synthesized TG under normal lipid absorption conditions [5]. In addition to the small intestine, the MG pathway is also active in adipose tissue and liver [6,7,8]. There are two identified MGATs (MGAT1 and MGAT2) in mice [9,10], as well as three MGATs (MGAT1, MGAT2, MGAT3) in humans [11,12,13]. Two identified DGATs, DGAT1 and DGAT2, function as the rate-limiting enzymes for TG biosynthesis [14,15]. Physical interactions between MGAT and DGAT enzymes promote TG synthesis in mammalian cells [16,17].

The G-3-P pathway begins with the acylation of G-3-P with acyl-CoA catalyzed by G-3-P acyltransferase (GPAT) to lysophosphatidic acid (LPA) [18,19]. LPA is further acylated by 1-acylglycerol-3-phosphate acyltransferase (AGPAT) producing phosphatidic acid (PA) [20]. Dephosphorylation of PA-by-PA phosphatase (PAP) yields DG as a precursor for the synthesis of TG [20]. The G-3-P pathway is responsible for the majority of de novo TG synthesis in most cells and tissues [21]. As the central intermediates in the G-3-P pathway, DG and PA are also precursors for the synthesis of amphipathic glycerophospholipids. PA is a precursor for the synthesis of CDP-DG that is further used for the synthesis of phosphatidylinositol (PI), phosphatidylglycerol (PG) and cardiolipin (CL). DG serves as a substrate for phosphatidylcholine (PC) and phosphatidylethanolamine (PE) synthesis by the CDP choline/ethanolamine pathways [21]. 

The mammalian glycerophospholipid acyltransferase family is characterized by a conserved acyltransferase domain (pfam01553) containing four conserved motifs [22,23]. Several acyltransferases including GPATs and AGPATs in the de novo synthesis of TG and glycerophospholipids share the pfam01553 domain [23,24]. In addition, the pfam01553 acytransferase family contains several lysophospholipid acyltransferase (LPLAT) enzymes that re-acylate lysophospholipids derived from partial hydrolysis of de novo synthesized phospholipids in a process termed Land’s cycle [24]. Transmembrane protein 68 (TMEM68) is a poorly characterized transmembrane protein that constitutes a distinct cluster in the pfam01553 glycerophospholipid acyltransferase family [23]. It localizes to the endoplasmic reticulum (ER) and is highly expressed in the brain [25]. However, the substrates of TMEM68 and its function in glycerolipid metabolism remain elusive. Recent in silico analysis showed that TMEM68 contained the conserved catalytic motif I with a HXXXXD signature [25], the most conserved motif among GPAT/AGPAT family enzymes [24,26]. The putative conserved active sites, His129 and Asp135, in the catalytic motif I of TMEM68 may constitute a catalytic dyad of acyltransferase [25]. In contrast, the substrate binding motif II and III in GPAT/AGPAT members are not well conserved in TMEM68 [24,25,26], suggesting TMEM68 may recognize and bind different substrates than GPAT and AGPAT enzymes. TMEM68 also contains the acyltransferase domain pfam03982, which defines the DG acyltransferase family consisting of DGAT2, MGATs and acyl-CoA wax acyltransferases [25]. A phylogenetic analysis showed that TMEM68 is closer related to DG acyltransferases than glycerophospholipid acyltransferases although the overall sequence similarity of TMEM68 with MGAT1 and MGAT2 is low [25]. In the current study, through overexpression and quantitative targeted lipidomic technologies, we found that TMEM68 increases cellular DG, TG and FFA levels and promotes LD formation. Moreover, we showed that TMEM68 overexpression also broadly regulates glycerophospholipid composition and expression of lipogenesis genes in mammalian cells. Finally, we demonstrated that TMEM68 displays MGAT and DGAT activities in vitro depending on the putative conserved active site residues of its acyltransferase motif. Our results provided first evidence that TMEM68 functions as acyltransferase and regulator of mammalian glycerolipid metabolism. 

## 2. Results

### 2.1. Overexpression of TMEM68 Promotes TG Accumulation

**Figure 1:**
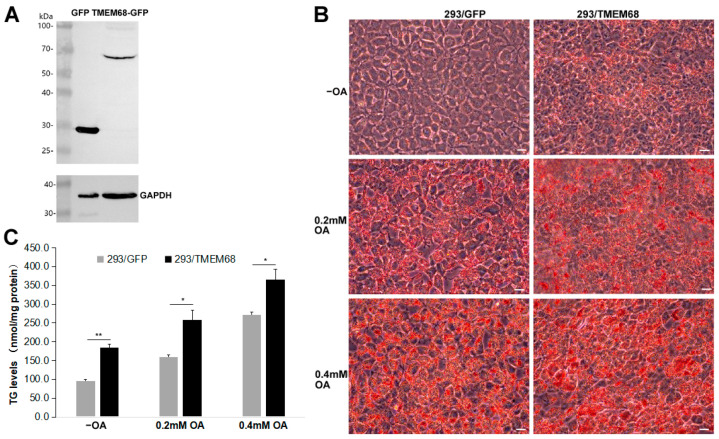
Overexpression of TMEM68 promotes TG accumulation. (**A**) The expression of GFP and TMEM68-GFP was detected by western blotting using an anti-GFP antibody in HEK293 cells that stably expressed GFP (293/GFP) and TMEM68-GFP (293/TMEM68), respectively. The expression of GAPDH was detected as loading control. Migration of molecular mass standard proteins is indicated left of the figure. (**B**) 293/GFP and 293/TMEM68 cells were loaded with 0.2 mM, 0.4 mM OA or not (−OA) for 12 h and then fixed LDs and subjected to ORO staining. Cell images were captured by using an Olympus IX53 inverted microscope. Images were representative of at least three experiments. Scale bars, 10 μm. (**C**) TG levels were quantified in 293/GFP and 293/TMEM68 cells in the presence or absence of 0.2 mM or 0.4 mM OA for 12 h. Data is presented as means ± SD. Asterisk indicates *p* values: * *p* < 0.05, ** *p* < 0.01, *n* = 4.

In order to explore the role of TMEM68 in lipid metabolism, HEK293 cells stably expressing TMEM68 fused with GFP (293/TMEM68) were generated. As shown in Figure 1A, immunoblotting analysis detected TMEM68-GFP in 293/TMEM68 cells but not in the control cells expressing GFP alone (293/GFP). Although TMEM68 is not a LD-binding protein, it is localized to the ER, the site of TG synthesis and LD formation [25]. Oil Red O staining of cells showed that overexpression of TMEM68 increased intracellular neutral lipid levels compared with GFP-expressing control cells in normal culture medium without oleic acid (OA) supplementation (Figure 1B). When cells were challenged with 0.2 mM or 0.4 mM OA to induce TG synthesis, Oil Red O staining also showed that the increased neutral lipid levels in TMEM68-expressing relative to control cells (Figure 1B). Quantification of TG levels revealed that TMEM68 overexpression increased the levels of TG 1.4–1.8 fold compared to control cells in the absence or presence of exogenous OA (Figure 1C). These results together indicate that TMEM68 overexpression promoted TG accumulation in mammalian cells.

### 2.2. The Increased TG Levels by TMEM68 Depends on the Conserved Acyltransferase Motif

**Figure 2:**
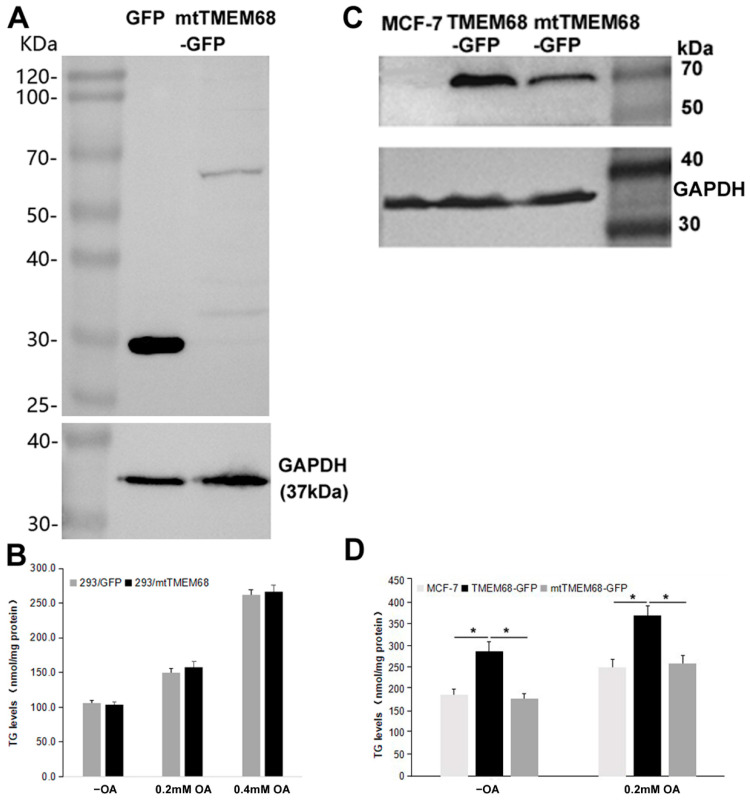
TG accumulation induced by TMEM68 overexpression depends on the conserved catalytic motif. A and C, the expression of GFP, TMEM68-GFP, mtTMEM68-GFP in HEK 293 (**A**) and MCF-7 (**C**) cells was detected by western blotting using an anti-GFP antibody. The expression of GAPDH was detected as the loading control and migration of molecular mass standard proteins is indicated. B and D, TG levels were quantified in HEK 293 (**B**) and MCF-7 (**D**) cells that transiently expressed GFP, TMEM68-GFP or mtTMEM68 cells in the absence or presence of OA for 12 h. Data is presented as means ± SD. Asterisk indicates *p* values: * *p* < 0.05, *n* = 4.

TMEM68 contains a conserved acyltransferase domain including a HXXXXD signature in catalytic motif I, the most conserved region among GPAT/AGPAT family enzymes [22,24,26]. The conserved D and H residues constitute a putative acyltransferase catalytic dyad [24,26]. We therefore asked whether the effect of TMEM68 on TG levels depends on its enzymatic activity. To this end, a mutant TMEM68 (mtTMEM68) containing H129A and D135N was constructed. The expression of mtTMEM68 in HEK293 cells did not increase the levels of TG compared with control cells expressing GFP under normal culture conditions and upon OA supplementation (Figure 2A,B). In addition, when TMEM68 and mtTMEM68 were expressed in parallel in human breast cancer MCF-7 cells (Figure 2C), TG levels in TMEM68-expressing but not in mtTMEM68-expressing cells were significantly elevated compared with non-transfected MCF-7 cells (Figure 2D). Therefore, mtTMEM68 did not affect cellular TG levels, suggesting that TMEM68 increased TG accumulation in an enzyme active sites-dependent manner.

**Figure 3:**
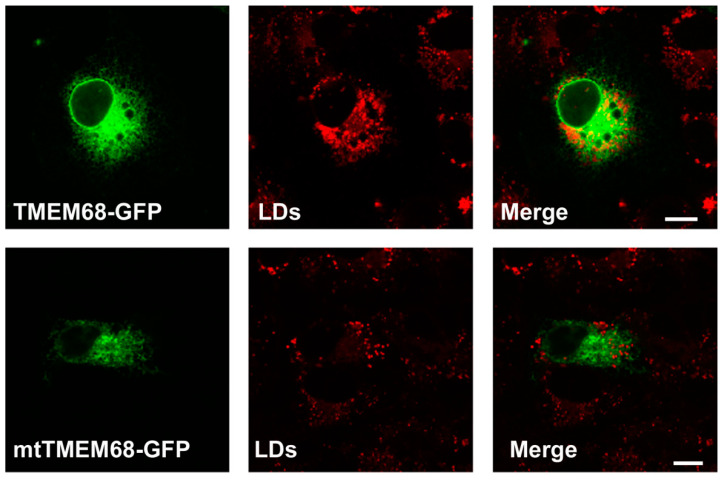
TMEM68 expression increases LDs content. MCF-7 cells were transfected with TMEM68-GFP and mtTMEM68-GFP, respectively, for 36 h, and then incubated with 0.2 mM OA for 12 h to induce LD formation. LDs were stained with HSC LipidTOX^TM^ Deep Red, and images were acquired by confocal fluorescence microscopy. Figures are representative of at least three experiments. Scale bars = 10 μm.

OA promotes the synthesis of TG that is stored in LDs. As shown in Figure 3, TMEM68 and mtTMEM68 tagged with GFP displayed a pattern resembling the ER in-challenged MCF-7 cells consistent with our previous results [25]. More LDs were accumulated in TMEM68-expressing cells after transient transfection compared with the less dispersed LDs in the adjacent cells lacking TMEM68 expression (Figure 3). Consistently, HEK293 cells stably expressing TMEM68 apparently contained more LDs as compared to 293/GFP cells even without OA supplementation (Supplementary Figure S1). In contrast, no apparent differences in LD size and number were observed between mtTMEM68-expressing cells and neighboring non-transfected cells (Figure 3).

### 2.3. TMEM68 Increases the Levels of TG, DG and FFA

**Figure 4:**
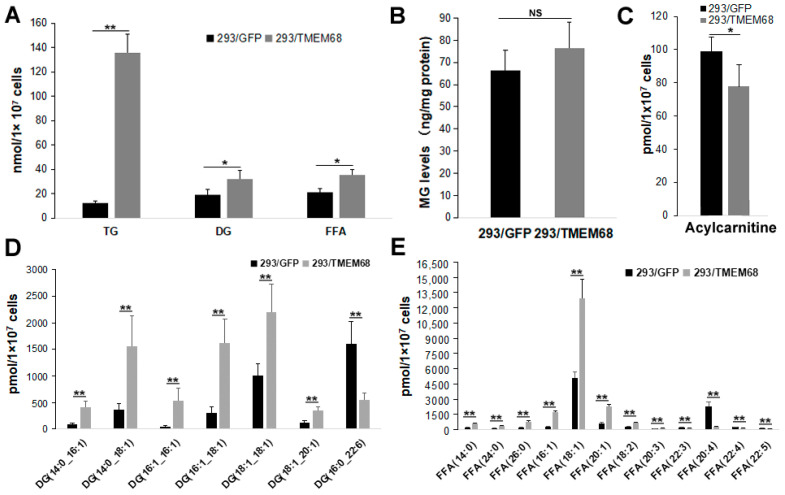
TMEM68 overexpression increases cellular TG, DG and FFA levels. TMEM68-overexpressing cells (293/TMEM68) and control cells (293/GFP) were collected for the quantification of various lipids by MetWare based on the AB Sciex QTRAP 6500 LC-MS/MS platform. (**A**) Levels of TG, DG and FFA in 293/GFP and 293/TMEM68 cells. (**B**) MG contents in 293/GFP and 293/TMEM68 cells as measured by ELISA. (**C**) Acylcarnitine level in 293/GFP and 293/TMEM68 cells. (**D**,**E**) Levels of DG (**D**), FFA (**E**) with differences ≥ 2.0 or ≤ 0.50 and VIP ≥ 1 between 293/GFP and 293/TMEM68 cells. Data are presented as means ± SD. NS, no significance. Asterisk indicated *p* values: * *p* < 0.05, ** *p* < 0.01, *n* = 6.

**Figure 5:**
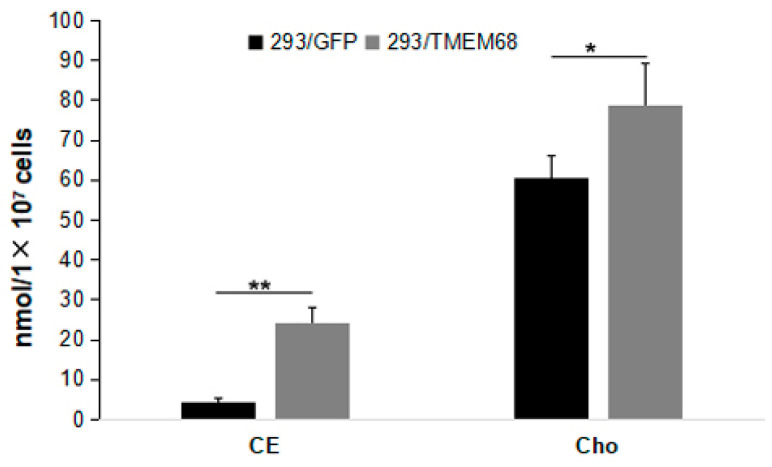
Quantification of cholesterol ester (CE) and cholesterol (Cho) levels in 293/TMEM68 and 293/GFP cells. Data were presented as means ± SD. Asterisk indicated *p* values: * *p* < 0.05, ** *p* < 0.01, *n* = 6.

Quantitative targeted lipidomic analysis was further performed to explore the influence of TMEM68 overexpression on intracellular lipids metabolism. A total of 810 lipids were detected, covering fatty acyl, glycerides, glycerophospholipids, sphingolipids, sterol esters, etc. Consistent with the previous results, total TG levels in 293/TMEM68 cells significantly increased (10-fold compared to 293/GFP control cells (Figure 4A)). The cellular levels of DG and FFA, the precursors of TG biosynthesis, also significantly increased upon overexpression of TMEM68 albeit to a lesser extent (Figure 4A). However, TMEM68 overexpression did not alter MG levels as measured by ELISA (Figure 4B). The acylcarnitine level decreased due to overexpression of TMEM68 (Figure 4C). In addition to TG, the levels of total cholesterol ester (SE), another form of neutral lipids stored in LDs, were also elevated by more than 5-fold in 293/TMEM68 cells relative to 293/GFP cells (Figure 5).

**Figure 6:**
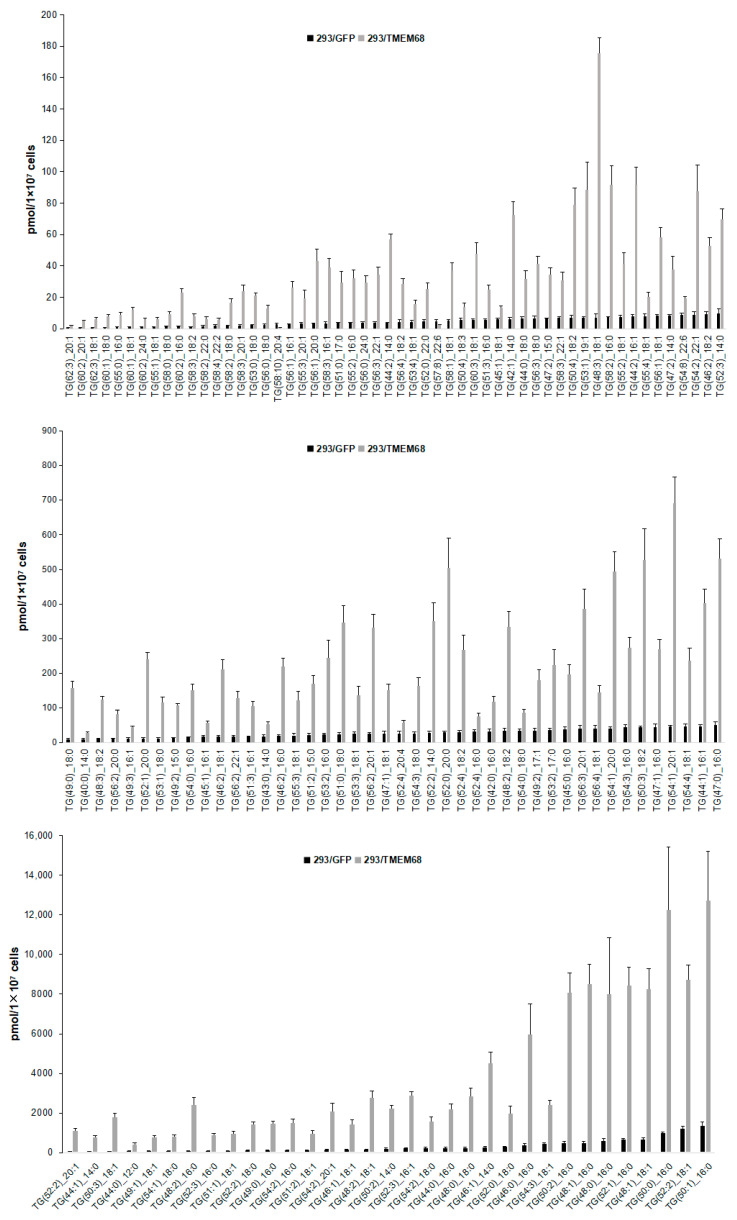
Comparing the levels of differentiated TG species between 293/GFP and 293/TMEM68 cells. TG levels were quantified by MetWare based on the AB Sciex QTRAP 6500 LC-MS/MS platform and the differentiated TGs were screened by VIP ≥ 1, FC ≥ 2.0 or FC ≤ 0.50. Data were presented as means ± SD. All *p* < 0.01, *n* = 6.

The lipid metabolites of 293/GFP and 293/TMEM68 cells were screened according to fold change (FC) ≥ 2.0 or FC ≤ 0.50 and VIP ≥ 1. 355 lipids were identified, of which 127 and 228 lipids were decreased and increased, respectively, in 293/TMEM68 cells (Supplementary Figure S2). Overexpression of TMEM68 increased the content of 6 DG species to more than 2-fold of control cells, all of which contained unsaturated palmitoleyl-, oleoyl- or both moieties. Conversely, the polyunsaturated DG (16:1_22:6) was decreased upon overexpression of TMEM68 (Figure 4D). 7 FFAs were increased, including saturated and monounsaturated FFAs, whereas 5 polyunsaturated FFAs containing arachidonic acid (FFA (20:4)) were reduced upon overexpression of TMEM68 (Figure 4E). A total of 131 TGs were screened out, most of which were increased with the exception of TG (58:10)_20:4 and TG(57:8)_22:6 (Figure 6). TG (48:2)_16:0 exhibited the highest relative increase in TMEM68-overexpressing cells (28-fold over control cells) and TG (50:1)_16:0 was the most abundant species (Figure 6).

**Figure 7:**
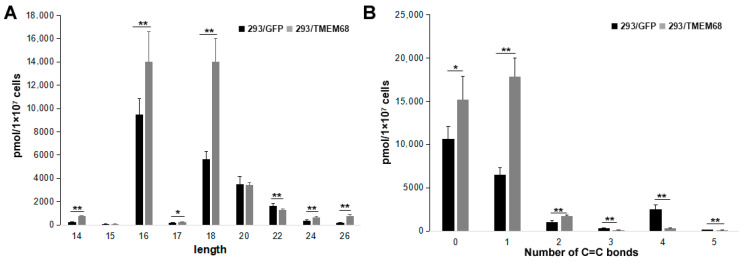
The levels of FFA with different length (**A**) and number of C=C bonds (**B**). Data were presented as means ± SD. Asterisk indicated *p* values: * *p* < 0.05, ** *p* < 0.01, *n* = 6.

Changes in FFA species were further characterized. The levels of long chain FFAs (14–20 carbon atoms) were increased in TMEM68-expressing cells compared with control cells. In contrast, very long FFAs (more than 20 carbon atoms) were decreased (Figure 7A). The content of saturated, mono- and di-unsaturated FFA species was increased by TMEM68 overexpression, whereas the content of polyunsaturated FFAs with more than two C=C bonds was reduced in 293/TMEM68 cells (Figure 7B).

### 2.4. TMEM68 Affects Glycerophospholipids Levels

**Figure 8:**
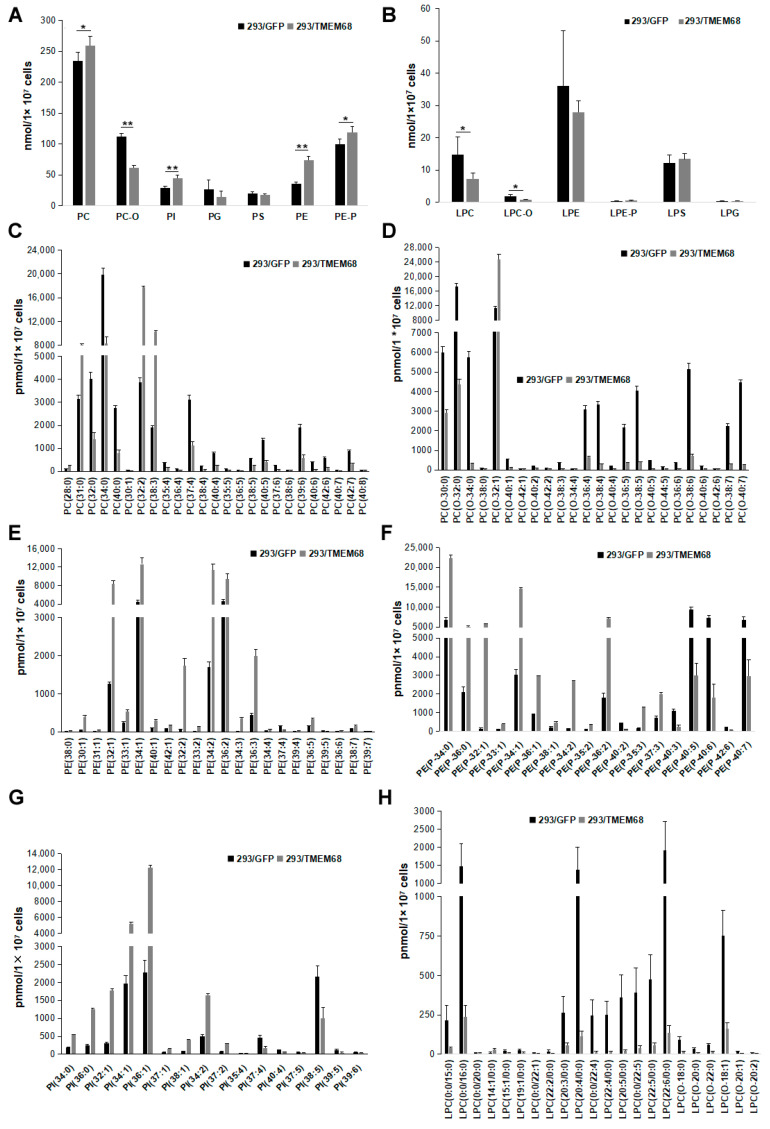
Glycerophospholipids levels were altered by TMEM68 overexpression. A and B, Comparison of total various glycerophospholipids (**A**) and lysophospholipids (**B**) levels between 293/TMEM68 and 293/GFP cells. Data were presented as means ± SD. Asterisk indicated *p* values: * *p* < 0.05, ** *p* < 0.01, n = 6. C-H, Comparing the levels of differentiated PC (**C**), PC-O (**D**), PE (**E**), PE-P (**F**), PI (**G**), LPC and LPC-O species (**H**) between 293/GFP and 293/TMEM68 cells. Differentiated lipids screened by VIP ≥ 1, FC ≥ 2.0 or FC ≤ 0.50 were shown. Data were presented as means ± SD. All *p* < 0.01, *n* = 6.

DG also serves as precursor for the synthesis of glycerophospholipids including PE and PC [21]. Quantitative targeted lipidomic analysis showed that TMEM68 overexpression increased the levels of PC and PE to more than 1.1- and 2-fold compared with control cells, respectively (Figure 8A). In addition, phosphatidylinositol (PI) levels were also significantly increased by more than 1.5-fold relative to control cells (Figure 8A). Ether glycerophospholipids are one of the major cell membrane lipid components and classified into two types: plasmalogen (1-O-alk-1′-enyl-2-acyl-, i.e., “P”) and alkyl-(1-O-alkyl-2-acyl-, i.e., “O”) glycerophospholipids depending on the substituents at the sn-1 position [27]. Naturally occurring ether-glycerophospholipids are mainly present as PC-O and PE-P [28]. The levels of PC-O and PE-P were reduced and increased, respectively, in TMEM68-expressing cells (Figure 8A). Moreover, TMEM68 overexpression reduced cellular lyso-PC (LPC) and LPC-O contents compared to control cells (Figure 8B). In addition, as a membrane lipid, the cholesterol levels were also increased due to overexpression of TMEM68 (Figure 5).

The total PC level showed a slight increase (10%) (Figure 8A). However, when PC species were analyzed in detail, 25 PC species were screened according to FC ≥ 2.0 or FC ≤ 0.50 and VIP ≥ 1. The PC (28:0), PC (31:0), PC (32:2) and PC (38:3) levels were increased, whereas levels of other PCs were reduced (Figure 8C). Thus, TMEM68 overexpression in HEK293 cells appeared to alter the relative abundance of certain PC species. The cellular levels of many PC-O species were reduced to less than 50% of control cells with the exception of PC (O-32:1) (Figure 8D). Most PE, PE-P and PI species levels were increased by TMEM68 overexpression to more than 2-fold of the control levels consistent with the increase in total PE, PE-P, and PI (Figure 8E–G). Overexpression of TMEM68 also reduced the contents of 21 LPC and LPC-O species to be less than those of control cells except an increased LPC (14:1/0:0) (Figure 8H). These data demonstrated that the expression of TMEM68 broadly influenced glycerophospholipids metabolism.

### 2.5. TMEM68 Exhibits MGAT and DGAT Activities

Although TMEM68 contains acyltransferase catalytic motif I, which is highly conserved among GPAT/AGPAT members, it is also related to the DG acyltransferase family [24,25]. Overexpression of TMEM68 increased cellular DG and TG levels (Figure 4). These data suggest that TMEM68 may exhibit enzymatic activities of MGAT and (or) DGAT.

**Figure 9:**
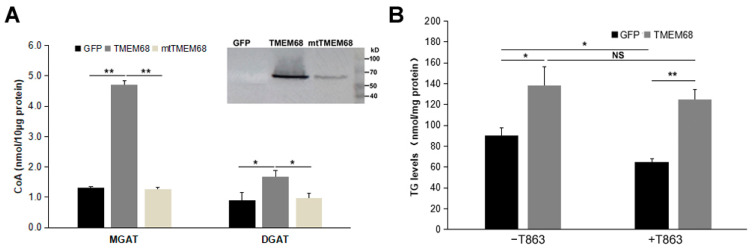
TMEM68 exhibites MGAT and DGAT activities. (**A**) Total membrane protein was extracted from 293/GFP, 293/TMEM68 and 293/mtTMEM68 cells. MGAT and DGAT activities of TMEM68 were measured by monitoring the released CoA from oleoyl-CoA in a TMEM68-mediated reaction containing 2-oleoyl-glycerol or 1-2-dioleoyl-*sn*-glycerol as the acceptor of oleoyl group. Immunoblot analysis of total membrane fraction isolated from HEK293 cells stably expressing GFP and TMEM68-GFP, transient expressing mtTMEM68. Migration of molecular mass standard proteins was indicated right of the figure. Data were presented as means ± SD. Asterisk indicated *p* values: * *p* < 0.05, ** *p* < 0.01, *n* = 3. (**B**) TG levels assays in HEK293 cells overexpressing GFP or TMEM68 in the absence or presence of T863. Cells were pretreated with T863 for 1 h or not, and then incubated with OA for 6 h. TG levels were quantified by an enzymatic triglyceride assay kit. Data were presented as means ± SD. NS, no significance. Asterisk indicated *p* values: * *p* < 0.05, ** *p* < 0.01, *n* = 3.

We therefore compared MGAT and DGAT activities of 293/TMEM68, 293/mtTMEM68, and 293/GFP cell homogenates by monitoring the release of CoA from an acyl-CoA in the presence of MG or DG [29,30]. The sulfhydryl (-SH) group of CoA can react with a coumarin derivative, 7-Diethylamino-3-(4-maleimidophenyl)-4-methylcoumarin (CPM), to form CPM-CoA. CPM-CoA is a readily detected fluorescent product at excitation and emission wavelengths of 355 and 460 nm, respectively [29,30]. When 2-oleoyl-glycerol was used as oleoyl acceptor to measure MGAT activity, cells expressing TMEM68 showed a ~3-fold increase in MGAT activity compared to GFP-expressing and mtTMEM68-expressing control cells (Figure 9A). Similarly, the enzymatic activity of DGAT was also enhanced by TMEM68 overexpression compared to GFP and mtTMEM68 when 1-2-dioleoyl-*sn*-glycerol was used as an oleoyl acceptor although to a lesser extent (Figure 9A). Importantly, expression of mtTMEM68 lacking putative active site residues did not increase acyltransferase activities in the presence of MG or DG when compared to GFP-expressing cells (Figure 9A). Thus, TMEM68 exhibits MGAT and DGAT activities dependent on its conserved active sites in vitro.

To further prove the DGAT activity of TMEM68, a DGAT1 selective inhibitor T863 was used to inhibit the catalytic activity of DGAT1 [29], because the expression of *DGAT1* gene was dozens of folds higher than *DGAT2* gene in HEK293 cells (Supplement Figure S3A). As shown in Figure 9B, compared with GFP-expressing cells, TMEM68-overexpressing cells showed more TG levels upon OA loading without T863 pretreatment. Treatment with T863 significantly reduced the cellular TG content in 293/GFP cells. In contrast, a significant reduction of TG levels was not observed in 293/TMEM68 cells, and more TG content was accumulated relative to 293/GFP cell after T863 incubation. This indicates that overexpression of TMEM68 increased cellular TG levels independent of DGAT1 activity. 

### 2.6. TMEM68 Overexpression Alters the Expression of Lipogenesis Genes

**Figure 10:**
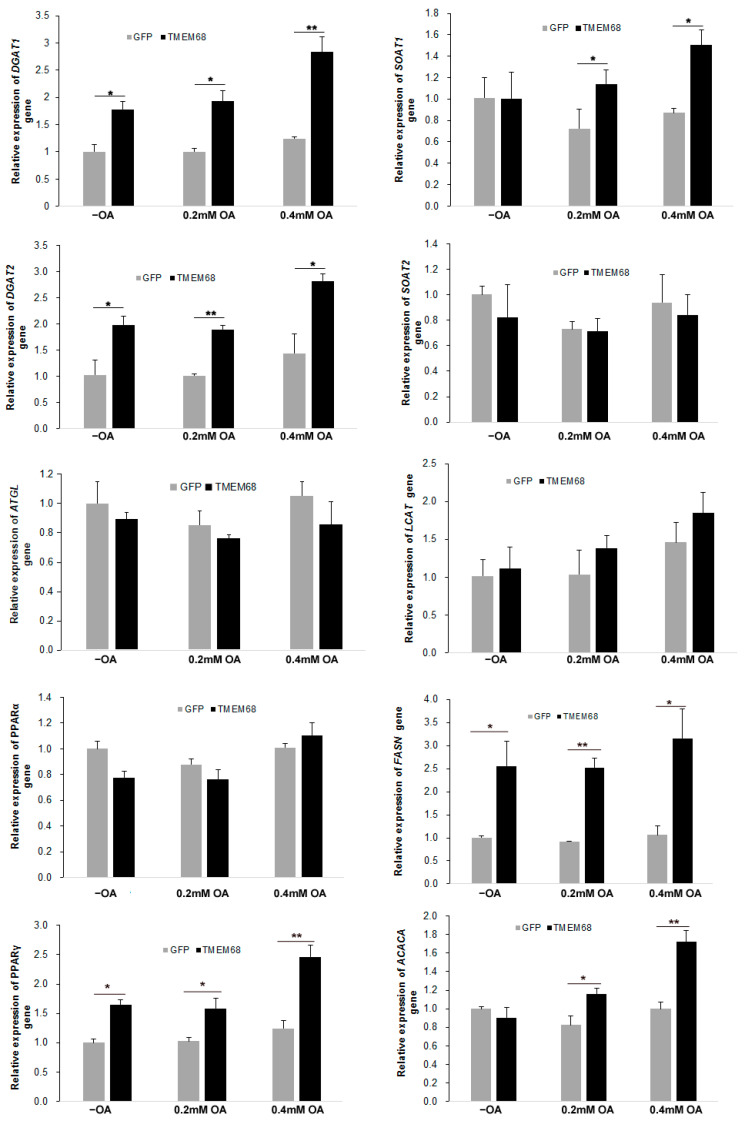
The expression of lipogenesis genes altered by TMEM68 overexpression. 293/GFP and 293/TMEM68 cells were incubated with OA at the indicated concentrations or not (−OA) for 12 h, and total RNA was then prepared and reverse transcribed to cDNA. mRNA levels were quantified by qPCR normalized to *β-actin* as an internal reference gene that were presented as fold change of 293/GFP cells without OA treatment. Data are presented as means ± SD. *p* values were shown by asterisk: * *p* < 0.05, ** *p* < 0.01, *n* = 6.

Given the broad effects of TMEM68 overexpression of glycerolipids levels, we next assessed expression of genes involved in lipogenesis by RT-qPCR. As shown in Figure 10, TMEM68 overexpression significantly increased the mRNA levels of *DGAT1* and *DGAT2,* the key enzymes for TG synthesis, in the absence and presence of OA supplementation. In contrast, the expression of *adipose triglyceride lipase* (*ATGL*), a key gene for TG mobilization, was largely unchanged. In addition, the expression of *peroxisome proliferator-activated receptor γ* (*PPARγ*), but not *PPARα*, was upregulated in 293/TMEM68 cells compared with control 293/GFP after OA loading (Figure 10). Because CEs levels were also elevated by overexpression of TMEM68, the expression of sterol-O-acyltransferase (SOAT) genes *SOAT1* and *SOAT2,* encoding for key enzymes of CE synthesis, was also quantified. Under normal culture condition, mRNA levels of *SOAT1* were not altered by TMEM68 overexpression. However, TMEM68 overexpression upregulated *SOAT1* mRNA levels upon OA supplementation. In contrast, TMEM68 overexpression did not affect the expression of the *SOAT2* gene. No changes in mRNA levels of *lecithin-cholesterol acyltransferase* (*LCAT*) gene were observed due to TMEM68 overexpression. Moreover, TMEM68 overexpression influenced the expression of genes involved in fatty acid synthesis. The mRNA levels of *fatty acid synthase* (*FASN*) were higher in 293/TMEM68 cells compared to 293/GFP cells in the presence and absence of exogenous OA and mRNA levels or *acetyl-CoA carboxylase α* (*ACACA*) were increased in 293/TMEM68 cells upon OA supplementation. Hence, overexpression of TMEM68 affected the expression of several genes related to TG, SE and FA synthesis.

## 3. Discussion

Acyltransferases catalyze de novo glycerolipid formation via the MG or G-3-P pathway and remodel glycerophospholipid species in the so-called Land’s cycle [24,26]. We previously identified TMEM68 as ER-localized transmembrane protein with a conserved pfam01553 acyltransferase family domain [25]. This family comprises GPAT, AGPAT and LPLAT enzymes with functions in the G-3-P pathway and the Land’s cycle [23]. TMEM68 also exhibits a phylogenetic relationship to the DG acyltransferase family, which includes DGAT2, MGATs and acyl CoA wax alcohol acyltransferases [25,31,32]. Here we show that overexpression of TMEM68 in mammalian cells elevates cellular MGAT and DGAT activities and promotes LD formation and TG synthesis, as well as alterations in glycerophospholipid composition and lipogenic gene expression. Mutagenesis of the conserved HXXXXD signature abrogates TMEM68-mediated increases in acyltransferase activities and renders TMEM68 unable to augment cellular TG. This suggests that TMEM68 indeed possesses acyltransferase activity and participates in TG synthesis via the MG rather than the G-3-P pathway of glycerolipid synthesis. 

TMEM68 is an ER-anchored transmembrane protein and does not localize to LDs [25], which resembles the localization of MGAT1-3 enzymes [9,11,13,33]. TMEM68 also displays some DGAT activity in vitro in addition to MGAT activity similar to MGAT1-3 [34,35]. TMEM68 overexpression increased not only cellular TG levels but also DG levels, further supporting a dual MGAT/DGAT-like function of TMEM68. Hence, the catalytic and cellular localization properties of TMEM68 closely resemble those of established MGAT enzymes. TMEM68 is highly expressed in mouse brain [25], a tissue that does not express detectable levels of MGAT1 or MGAT2, but exhibits measurable MGAT activity [9,10]. In addition to DG and TG, FFA levels were also increased by TMEM68 overexpression, whereas acylcarnitine level was decreased, which suggested that more FFA was channeled to TG synthesis and less FFA to be oxidized for energy.

Expression of TMEM68 increased mRNA levels of several key regulators of lipogenesis, including both DGAT genes, *DGAT1* and *DGAT2*. Conversely, TMEM68 overexpression did not significantly alter the expression of the *ATGL* gene, which initiates the hydrolysis of TG to DG in lipolysis [36]. In addition to DGATs, TMEM68 expression also increased mRNA levels of *ACCA* and *FASN*, two key enzymes of de novo FA synthesis, which catalyze carboxylation of acetyl-CoA to malonyl-CoA and condensation of acetyl-CoA and malonyl-CoA to FAs, respectively [37]. TMEM68 overexpression also increased the expression of the *PPARγ* gene which is a major regulator of lipogenesis [38], but not of the *PPARα* gene. Taken together, these transcriptional changes likely impact cellular FA and TG synthesis rates and may thus account, in part, for the observed increases in cellular neutral lipids and FA levels observed upon TMEM68 overexpression. Along these lines, TMEM68 did not only augment TG storage but also increased cellular CE levels that correlated with an increase in the mRNA levels of *SOAT1*, which was highly expressed relative to *SOAT2* gene in HEK293 cells (Supplementary Figure S3B). These observations make it difficult to unequivocally assign increased TG and LD formation to TMEM68 MGAT/DGAT activities. However, TMEM68-related increases in cellular TG levels were also observed after pharmacological inhibition of DGAT1 arguing against the notion that TMEM68 indirectly regulates TG via modulating DGAT1 expression.

Lipidomic analysis revealed that overexpression of TMEM68 did not only affect cellular neutral lipids but had a profound impact on cellular glycerophospholipid composition as well. In particular, TMEM68 expression markedly increased total PI and PE levels and decreased LPC and PC-O levels. Although total PC and PE-O levels were less affected by TMEM68 expression, the molecular composition of these phospholipids was altered. The synthetic neutral lipids, including TG and SE, are stored in the core of LDs, whose surface is surrounded by a phospholipid monolayer [39]. The phospholipid monolayer is composed by hundreds of phospholipid molecules, of which PC is the most abundant lipid class [40]. In addition to PC, PE and PI are also the components of LD phospholipid monolayer [41]. Overexpression of TMEM68 promoted the accumulation of TGs and the formation of more LDs. The increase of phospholipids such as PC, PE and PI is an adaptive mechanism for new lipid synthesis in TMEM68-expressing cells. In addition, highly polyunsaturated PC and PE-O species were depleted, whereas specific species with lower saturation degree were increased upon expression of TMEM68. Polyunsaturated PI, DG, and FA were also reduced upon TMEM68 expression. This suggests a role of TMEM68 in channeling specific FA species into glycerolipids, although the exact pathways implicated in those alterations remain to be elucidated. 

In summary, our study provides a first characterization of the intrinsic acyltransferase activities of the poorly characterized TMEM68. Our data suggest that TMEM68 harbors MGAT/DGAT activity and participates in the MG pathway of TG synthesis. Nevertheless, the complex alterations in the membrane lipidome induced by TMEM68 expression point to functions of TMEM68 in glycerolipid metabolism beyond its MGAT/DGAT-like activity that remain to be further elucidated. 

## 4. Material and Methods

### 4.1. Materials

Human embryonic kidney HEK293 cells and breast cancer MCF-7 cells were purchased from Shanghai Cell Center of Chinese Academy of Sciences (Shanghai, China). Plasmid pEGFP-N3 was purchased from Clontech (Mountain View, CA, USA). Transfection reagent Lipofectamine 3000 and HCS LipidTOX™ Deep Red neutral lipid stain were purchased from Thermo Fisher Scientific (Waltham, MA, USA). OA, 7-Diethylamino-3-(4-maleimidophenyl)-4-methylcoumarin (CPM) were obtained from Sigma-Aldrich (St. Louis, MO, USA). 2-oleoyl-*sn*-glycerol (2-MG), 1-2-dioleoyl-*sn*-glycerol (DG), Coenzyme A (free acid) and 18:1 (n9) Coenzyme A were obtained from Avanti Polar Lipids (Alabaster, AL, USA). The Mut Express Ⅱ Fast mutagenesis Kit v2 was purchased from Vazyme Biotechnology (Nanjing, China). The E1025 triglyceride assay kit was purchased from Applygen Technologies (Beijing, China). DGAT1 inhibitor T863 was purchased from MedChemExpress (Shanghai, China). Mouse anti-GFP, anti-GAPDH monoclonal antibodies, goat anti-mouse IgG HRP and enhanced chemiluminescence (ECL) reagents were obtained from Beyotime Biotechnology (Shanghai, China).

### 4.2. Plasmids Construction 

To generate mutant TMEM68 (mtTMEM68), the putative conserved acyltransferase catalytic sites H129 and D135 were changed to A and N, respectively, the forward and reverse primers, 5’AGGAGCCATCCCCATAAACTTTTACTACTTCATGGCTAAAATTTTCA3’ and 5’TTATGGGGATGGCTCCTGCATAAAAAATTATAAGTGCAGCTCCTTCT3’, were synthesized to amplify DNA using pTMEM68-GFP as the template, according to the relevant manual of Mut Express Ⅱ Fast Mutagenesis Kit v2. The total PCR volume contained 25 µL 2 × Max Buffer, 1 µL 10 mM dNTPs, 2 µL 10 µM forward and reverse primers, 2 µL pTMEM68-GFP (80 ng) and 1 µL Phanta^®^ Max Super-Fidelity DNA Polymerase and 19 µL H_2_O. Amplification procedure is 95 °C for 3 min to pre-denature, then 95 °C for 15 s; 62 °C for 15 s, and 72 °C for 5 min to amplify production for 30 cycles, and 5 min to extension. The PCR amplification product was digested with Dpn I for 1.5 h at 37 °C, which was recombined by Exnase II for 0.5 h at 37 °C. The recombined construct was transformed into DH5a strain. The mutant plasmid mtTMEM68-GFP was sequenced to confirm the presence of the desired mutations. 

### 4.3. Cell Culture, Transfection and Treatment

HEK293 cells were cultured in DMEM supplemented with 10% fetal bovine serum (FBS), 100 units/mL penicillin and 100 µg/mL streptomycin in a 37 °C incubator with 5% CO_2_. MCF-7 cells were cultured in DMEM containing 10% FBS, 1% non-essential amino acids, 0.01 mg/mL recombinant insulin, 100 units/mL penicillin and 100 µg/mL streptomycin in a 37 °C incubator with 5% CO_2_. Cell transfection was performed using Lipofectamine 3000 reagent. After transfection for 48 h, HEK293 expressing GFP and TMEM68-GFP were incubated with 600 µg/mL G418 for 2–3 weeks to generate stable expression cell clones. The positive cell clones were identified with western blotting and maintained in DMEM medium containing 200 µg/mL G418. GFP- and TMEM68-overexpressing HEK293 cells were pretreated with DGAT1 inhibitor T863 at a concentration of 10 µM for 1 h, followed by incubation with 0.2 mM OA supplemented with fat-free BSA for 6 h.

### 4.4. Western Blotting

Total protein samples were prepared in RIPA Buffer plus 1 mM phenylmethanesulfonyl fluoride (PMSF) by sonication, and then centrifuged with 15,000× *g* for 15 min at 4 °C to pellet the cell debris. The supernatant protein concentration was assayed with BCA Protein Assay Kit (Beyotime Biotechnology, Shanghai, China). The supernatant was mixed with 5 × SDS loading buffer and boiled for 5 min. All the samples were subjected to SDS/PAGE, transferred to nitrocellulose filters and subjected to immunoblotting analysis using anti-GFP antibody (1:1000 dilution) as described previously [42]. 

### 4.5. Oil Red O Staining 

To induce TG synthesis and LD formation, cells were loaded with 0.2 mM, 0.4 mM OA complexed to fat-free BSA for 12 h. After removing medium and washing with PBS, cells were fixed with 4% paraformaldehyde in PBS for 10 min at room temperature and then stained using Oil Red O stain kit (Solarbio, Beijing, China). Cell images were acquired using an Olympus IX53 inverted microscope. 

### 4.6. Measurement of TG Levels

Briefly, cells were washed with PBS for two times and harvested, and then dissolved in 100 µL lysis buffer per 1 × 10^6^ cells. After brief centrifugation, the protein concentration of the supernatant was assayed by BCA protein assay kit (Beyotime Biotechnology, Shanghai, China). The supernatant was boiled at 70 °C for 10 min and centrifuged with 2000 rpm for 5 min. The standard curve was drawn and TG levels were measured by the triglyceride assay kit E1025 (Applygen, Beijing, China).

### 4.7. Confocal Fluorescence Microscopy

For detection of LDs, cells were seeded in 12-well plate mounted onto cover slips and transfected as described above. After transfection for 36 h, the cells were loaded with 0.2 mM OA for 12 h, and then washed three times with 1× PBS and fixed with 4% paraformaldehyde (PFA) for 30 min at room temperature. LDs were stained with HCS LipidTOX^TM^ Deep Red (1:1000 in PBS) for 30 min. Fluorescent images were acquired by confocal scanning microscopy with a Leica SP5 confocal microscope equipped with a Leica HCX 63 × 1.4 NA oil immersion objective. GFP was excited at 488 nm, and emission was detected between 500 and 530 nm. HCS LipidTOX^TM^ Deep Red was excited at 633 nm, and emission was detected between 650 and 700 nm. All the presented experiments were repeated independently at least 3 times.

### 4.8. Quantitative Targeted Lipidomic Analysis

HEK 293 cells stably expressing GFP and TMEM68 were used for quantitative targeted lipidomics experiments. All 1 × 10^7^ cells were collected in one tube and each set contained 6 tubes (repeats). The targeted lipidomics experiments were carried out by METWARE (Wuhan, China) (http://www.metware.cn/, accessed on 15 May 2021). Lipids’ contents were detected by METWARE database based on the AB Sciex QTRAP 6500 LC-MS/MS platform. The fold change (FC) of the nonparametric test and variable importance in projection (VIP) calculated on the basis of the orthogonal partial least squares discriminant analysis (OPLS-DA), were used in combination to screen the differential lipid metabolites. Then, differential lipid metabolites between 293/GFP and 293/TMEM68 cells were screened according to the standard cut offs of VIP ≥ 1, FC ≥ 2.0 or FC ≤ 0.50 [43]. 

### 4.9. Quantification of MG Levels

Cells were washed with PBS twice and then lysed with RIPA buffer containing PMSF, which were centrifuged with 15,000× *g* for 5 min at 4 °C. The supernatant protein concentration was assayed with BCA Protein Assay Kit (Beyotime Biotechnology, Shanghai, China). MG levels in were quantified by human monoacylglycerol ELISA kit based on MG standards (Shanghai Zhenke Biotechnology Co., LTD, Shanghai, China). The supernatant was incubated with HRP-conjugated anti-MG antibody at 37 °C for 60 min. The reaction solution was discarded and washed five times. 50 μL of substrate A and B were added into each well and incubated at 37 °C for 15 min in the dark. Lastly the reaction was terminated by 50 μL of termination solution. OD450 value was measured within 15 min. 

### 4.10. Fluorescence-Based MGAT and DGAT Activities Assay

MGAT and DGAT activities of TMEM68 were assayed by monitoring the released CoA from a TMEM68-mediated reaction as previous description with a brief modification [29,30]. Total membrane protein was extracted from 293/GFP and 293/TMEM68 cells and mtTMEM68 transient expressing cells using the Membrane and Cytosol Protein Extraction kit (Beyotime Biotechnology, Shanghai, China) and protein concentration was determined using the BCA assay kit according to the manufacturer’s instructions. The enzyme activity assay was developed in the 96-well format. Briefly, the assay was carried out in a total volume of 100 µL containing 100 mM Tris-HCl, pH7.4, 200 μM 2-oleoyl-glycerol or 1-2-dioleoyl-*sn*-glycerol, 100 μM oleoyl-CoA and 10 μg membrane protein. The reaction carried out for 30 min after the addition of membrane protein extraction. Then the reaction was terminated by 0.1% SDS at a final concentration with an additional 30 min incubation. Lastly, 40 µL of CPM at 1000 µM concentration was added to the reaction for 30 min incubation at room temperature. The fluorescent signal was detected at Ex. 355 nm, Em. 460 nm by a Thermo Scientific- Varioskan™ LUX. A standard curve of CoASH (ranging from 0 to 400 µM of CoA) was generated together with each assay.

### 4.11. RNA Extraction and RT-qPCR 

Total RNA was extracted using EASYspin plus RNA extraction kit (Aidlab technology, Beijing, China) and then transcribed to cDNA with ueIris RT mix with DNase (All-in-One) kit (Yuheng Biotechnologies, Suzhou, China) according to the manufacturer’s instructions. Real-time quantitative PCR (qPCR) was performed with ChamQ SYBR qPCR Master Mix (Vazyme biotechnology, Nanjing, China) on a QuantStudio 3 Real-Time PCR System. The primers used were shown in Table 1. To account for differences in cell numbers, all cycle threshold (Ct) values of sample replicates were normalized to those of *β*-*actin* as reference gene. Relative mRNA levels were quantified with the ΔΔCt method [44].

### 4.12. Statistical Analysis

Data were generally expressed as mean ± standard deviation (SD) values. Groups of data were compared by one-way ANOVA analysis using the T-TEST method with two tails and two-sample heteroscedasticity. A difference between means was considered significant at *p* < 0.05.

## 5. Conclusions

In summary, we found that TMEM68 harbors intrinsic MGAT and DGAT activities and regulates cellular TG content and glycerolipid composition in an activity-dependent manner. TMEM68 may contribute to MGAT activity in the mouse brain, which expresses high mRNA levels of TMEM68 but undetectable mRNA levels of canonical MGAT1 and MGAT2 enzymes [9,10,25]. TMEM68 acyltransferase activity may play an important role in the homeostasis of TGs and other glycerolipids in the central nervous system.

## Figures and Tables

**Table 1:** Primers used in qPCR experiment.

Primer Name	Primer Sequence (5’ to 3’)
hATGLF	GAGATGTGCAAGCAGGGATAC
hATGLR	CTGCGAGTAATCCTCCGCT
hDGAT1F	GGTCCCCAATCACCTCATCTG
hDGAT1R	TGCACAGGGATGTTCCAGTTC
hDGAT2F	ATTGCTGGCTCATCGCTGT
hDGAT2R	GGGAAAGTAGTCTCGAAAGTAGC
hFASNF	AAGGACCTGTCTAGGTTTGATGC
hFASNR	TGGCTTCATAGGTGACTTCCA
hACACAF	TCACACCTGAAGACCTTAAAGCC
hACACAR	AGCCCACACTGCTTGTACTG
hPPARαF	TTCGCAATCCATCGGCGAG
hPPARαR	CCACAGGATAAGTCACCGAGG
hPPARγF	TACTGTCGGTTTCAGAAATGCC
hPPARγR	GTCAGCGGACTCTGGATTCAG
hSOAT1F	GGTGCGCTCTCACAACCTTT
hSOAT1R	GAGGTGCTCTCAAATCCTTCG
hSOAT2F	ATGGAAACACTGAGACGCACA
hSOAT2R	GGTAGGATTGTATAGCCTCCCG
hLCATF	AAGGACCGCTTTATTGATGGC
hLCATR	ATGCGAGAGGGAAACATCCAG
hactinF	CATGTACGTTGCTATCCAGGC
hactinR	CTCCTTAATGTCACGCACGAT

## Data Availability

Not applicable.

## Supplementary Materials

The following supporting information can be downloaded at: https://www.mdpi.com/article/10.3390/ijms24032012/s1.
