# Supplementary material for: Transmembrane Protein 68 Functions as an MGAT and DGAT Enzyme for Triacylglycerol Biosynthesis
Source: Int J Mol Sci. 2023 Jan 19;24(3):2012. doi: 10.3390/ijms24032012 (PMC9916437; doi:10.3390/ijms24032012)
Supplement: Supplementary file 1 [file ijms-24-02012-s001.zip › ijms-2151582-supplementary.pdf]

International Journal of
Molecular Sciences

MDPI

Supplementary material

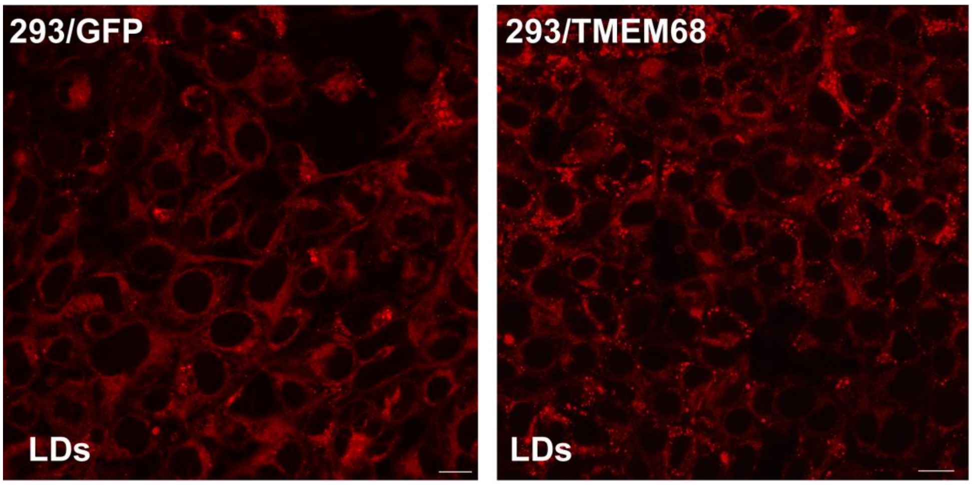

Figure S1. LDs staining in HEK 293 cells expressing TMEM68 and control GFP. HEK 293 cells expressing TMEM68 (293/TMEM68) and its control cells (293/GFP) were cultured without OA loading. LDs were stained with HSC LipidTOX™ Deep Red and visualized by confocal fluorescence microscopy. Figures were representative of at least three experiments. Scale bars, 10 μm.

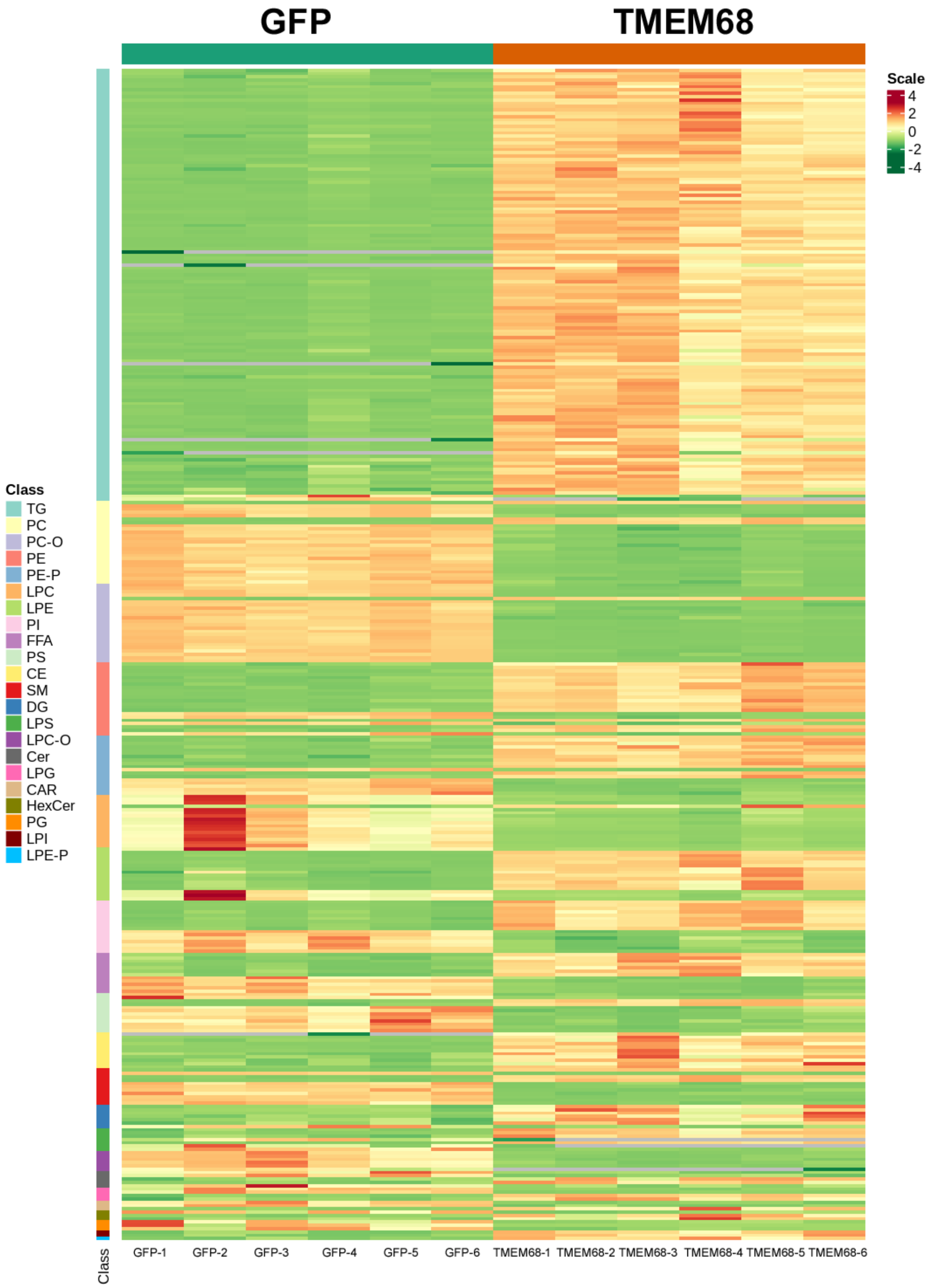

Figure S2. Heatmap of the differentiated lipid metabolites between 293/TMEM68 and 293/GFP cells. The differentiated lipid metabolites were screened according the standard, FC≥ 2.0 or FC ≤ 0.50, VIP ≥ 1.

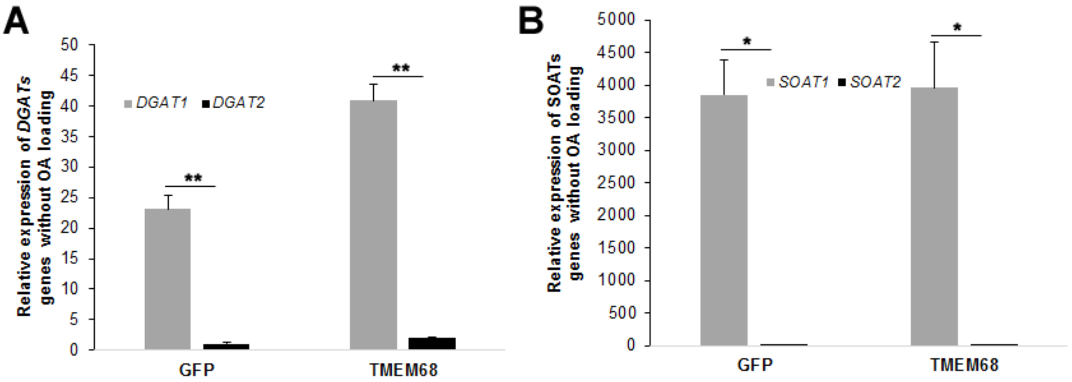

Figure S3. The expression of *DGATs* (A) and *SOATs* (B) genes. Total RNA from 293/GFP and 293/TMEM68 cells was prepared and then reverse transcribed to cDNA. mRNA levels were quantified by qPCR normalized to β-actin as an internal reference gene, and were presented as fold change of 293/GFP cells. Data are presented as means ± SD. *P* values were shown by asterisk: * $P < 0.05$, ** $P < 0.01$, n = 6.
